# The challenges of cardiac surgery for African children

**DOI:** 10.5830/CVJA-2012-013

**Published:** 2012-04

**Authors:** Ana Oh Mocumbi

**Affiliations:** Instituto do Coração, Maputo, Mozambique

**Keywords:** paediatric cardiology, cardiac surgery, Africa

## Abstract

**Abstract:**

In Africa the specific pattern of cardiovascular diseases and lack of adequate measures for disease prevention and control result in the frequent need for open-heart surgery for the management of complications of cardiomyopathies in children. Several strategies and innovative ways of providing cardiovascular surgical care in African countries have been used, from agreements to send patients overseas, to programmes for the creation of local services to provide comprehensive care locally.

This article attempts to outline the challenges faced by underdeveloped countries in Africa wanting to embark on programmes of cardiac surgery and the need for several sectors of society to play a role in the process. It discusses issues related to the establishment of centres performing cardiac surgery in Africa, describes the treatment of congenital heart disease, and reviews the aspects of management of conditions highly prevalent in or mostly confined to this continent, such as rheumatic heart valve disease and endomyocardial fibrosis.

## Abstract

Adequate measures for disease prevention and control are difficult to implement in Africa due to low levels of literacy, and poor sanitation and governance. On the other hand, timely diagnosis and management of cardiovascular diseases is hampered by shortage of qualified human resources and financial constraints. For these reasons, surgery is frequently needed for the management of cardiac conditions in African children.

Cardiac surgery imposes a huge burden on limited healthcare resources and is therefore not available in most sub-Saharan countries. Where it is available, surgery is performed in small numbers due to financial constraints and shortage of human resources. Therefore, several countries are running collaborative programmes between local institutions and teams from Europe and America, mostly sponsored by local or international non-governmental organisations.

We review here the challenges of surgical management of conditions that affect predominantly children, which are either highly prevalent in or confined to the African continent, namely rheumatic heart valve disease (RHVD), congenital heart disease (CHD) and endomyocardial fibrosis (EMF).

## Rheumatic heart valve disease

Although preventable, RHVD is still a very common condition in Africa, affecting young people and progressing quickly to severe forms that need heart valve surgery.^1^ Patients usually present in a poor general and nutritional condition, increasing the operative risk, and multiple lesions are frequent, requiring sub-optimal solutions on many occasions.^2^ Valve replacement presents a dilemma due to the generalised lack of adequate facilities for anticoagulation. Of major importance also are the issues related to the logistics of rheumatic fever prophylaxis, prevention of infective endocarditis and contraception in poor and remote areas. These factors lead to results that suggest that surgery for rheumatic heart valve disease in children is essentially a palliative procedure.^2^

In African studies researching the epidemiology of RHVD, the most frequent lesion is pure mitral valve regurgitation.^3^ This is in contrast with data from the developed world, where haemodynamically severe rheumatic mitral valve disease generally presents in the fourth decade or later, with thickened valve leaflets usually manifesting as mitral stenosis with or without concurrent regurgitation.^4^ The aortic valve is the second most frequently affected, but tricuspid regurgitation is also very common due to late diagnosis, when severe mitral and/or aortic disease cause pulmonary hypertension and dilatation of the right ventricle.

The choice of surgical technique is challenging and the results of valve repair performed during the active phase of rheumatic heart disease preclude a durable long-term result,^4^ highlighting the importance of correct pre-operative evaluation and preparation. Mitral valve repair is advised whenever technically feasible to maximise survival and reduce morbidity, even considering that good surgical results are inversely related to age,^5^ and that there is a risk of early re-operation due to inadequate secondary prophylaxis. On the other hand, while it has been recognised that good results can be achieved in children without the use of annuloplasty rings,^6^ the advantages of this strategy need to be confirmed in the long term.

Regarding treatment of the aortic valve, the Ross procedure has advantages in young and deprived populations since use is made of autologous and living tissue, there is excellent haemodynamics, allowance is made for growth and there is no need for anticoagulation. However, it also has some drawbacks, namely the need for homography in the pulmonary position and the fact that it is a technically demanding procedure.

Some authors feel that the Ross operation it is not suitable for young patients with RHVD. Although there is no sudden dilatation in children, some degree of aortic regurgitation appears in up to 18.3% after 2.4 years of follow up, and it is known that young age and associated mitral valve disease are significant risk factors for autograft failure.^7^ Finally, rheumatic involvement of the autograft has been reported, leading some to contraindicate the Ross operation in young patients with rheumatic heart valve disease.^8^,^9^ However, we feel that in Africa, it still the most valuable option for management of aortic disease in females. The double autograft, also considered in poor settings due to shortage of homographs, needs high levels of surgical skill and increases the operative risk.

## Congenital heart disease

There is a large underserved population of children with congenital heart disease in Africa, since most paediatric services are oriented to the diagnosis and management of endemic infectious diseases, and there is a shortage of trained personnel capable of diagnosing congenital heart defects, resulting in late diagnosis, usually in the presence of heart failure, pulmonary hypertension and severe polycythaemia. The number of facilities for cardiac surgery is also small and, as a result, there is a wide range of native abnormalities.^10^

Malformations ranked as the second most common form of heart disease, with a frequency of 26% among black patients of all ages in hospital-based studies, which also revealed that congenital cardiac defects were the dominant conditions rather than rheumatic or other acquired heart diseases.^10^ In this study, the mean age at referral to a paediatric cardiologist was high and a pattern of late presentation was found with under-representation of lesions that have a high mortality in infancy, suggesting that a significant number of patients miss the opportunity to have optimal surgical intervention.

Several paediatric hospital series show the importance of CHD in Africa. These conditions ranked first, with a frequency of 53% in a series from South Africa that considered only children aged 15 years or under,^11^ and are responsible for one-quarter of the cases of heart failure in Ibadan.^12^ The predominant lesions are ventricular septal defect, tetralogy of Fallot and patent ductus arteriosus.^10^,^11^

In Africa, surgery for CHD is frequently performed in adults or adolescents, in whom the operative risks are increased and related to myocardial fibrosis and irreversible pulmonary changes. The main reasons for late surgery are late diagnosis, time delay between the diagnosis and actual surgery, loss to follow up, and refusal of surgery earlier in life.

The choice between complete repair and two-stage ‘palliative + corrective’ procedures in underdeveloped countries may be influenced by socio-economic factors affecting the physical condition of the patient. Palliation by pulmonary artery banding, atrial septectomy or a systemic–pulmonary shunt is still preferable in those conditions in which total correction in infancy carries a high risk or is not feasible. On the other hand, palliative procedures may constitute a way of selecting those patients in whom eventual complete correction would be justified.^13^ However, if inadequately performed, pulmonary artery band and systemic–pulmonary shunt may adversely affect outcome and demand further aggressive management prior to definitive repair.^14^

## Endomyocardial fibrosis

EMF is a disease of unknown aetiology, characterised by fibrosis and thickening of the mural endocardium and valvular apparatus, causing restriction to ventricular filling and severe atrioventricular valve dysfunction. The disease has several forms and can be classified haemodynamically as predominantly restrictive when it affects mainly the mural endocardium, predominantly valvular when the subvalvular apparatus is severely affected, or mixed when both restrictive and valvular lesions are present. There is no effective treatment for EMF. Surgery is indicated for patients in NYHA class III/IV but is technically very demanding and has been associated with high morbidity and mortality, especially when predominantly murally affected.

The initially described Dubost technique^15^ has been evolving to reduce the complications associated with valve replacement and radical endocardectomy, mostly low-cardiac output syndrome and complete atrio-ventricular block. An atrial approach is the most frequently used but offers poor visualisation of the apex and lateral wall of the left ventricle. Therefore apical ventriculotomy or a transaortic–transatrial approach may be needed when complete endocardial resection is required in those regions.^16^

The post-operative period demands intensive-care management in patients with poor nutritional status and longstanding heart failure. The early mortality was initially around 20% but has now been reduced to 10%.^17^-^19^ This is mainly caused by incapacity to wean from cardiopulmonary bypass, low-cardiac output syndrome, cerebral embolism, arrhythmias, renal failure and pulmonary embolism.^17^,^20^,^21^ In the immediate post-operative period, re-interventions may be needed as a rescue procedure in patients with right heart failure and low-cardiac output syndrome, to relieve persistent pericardial effusion with repetitive tamponade and to implant partial cavo–pulmonary shunts.^22^,^23^ Most patients have dramatic clinical improvement with regression of ascitis and congestive failure, as well as improvement in quality of life, although the ultimate prognosis is probably not altered.^24^ However, mortality remains high during the first two post-operative years, reaching 13%.^20^

Good long-term results may be obtained with early diagnosis and timely intervention before shrinkage of the ventricle occurs. Promising results have been obtained in terms of restoration of both structural and functional changes, with better understanding of the pathophysiology and the use of a new tailored approach for relief of right ventricular cavity obliteration.^25^ Although there was no evidence of recurrence over a short follow-up period, further research and longer follow up is needed, since recurrence of endocardial fibrosis has been reported in a Brazilian series.^26^

## The challenges for sub-Saharan Africa

The African paediatric population is extremely underserved by paediatricians in general and by paediatric cardiologists in particular, most working in the major referral units in urban areas. Families have therefore to travel long distances in search of medical care for their children with heart disease. The state-run health infrastructures are mostly directed at prevention and treatment of the major endemic diseases, such as malaria, tuberculosis, leprosy, HIV/AIDS, parasitic infections and diarrhoeal diseases.

Due to lack of financial and human resources, both interventional cardiology and open-heart surgery have been introduced in Mozambique, Kenya, Sudan, Ethiopia, Senegal and Nigeria through collaboration programmes between local institutions and non-governmental organisations from Europe and North America.

Comprehensive paediatric cardiovascular services are needed in Africa. These should include research aimed at understanding the mechanisms underlying conditions geographically restricted to this continent, targeting country-specific conditions, and investigating the role of toxins and traditional practices in the pathogenesis of cardiovascular conditions. Apart from providing the means to enable the clinical services to achieve excellence, research provides important tools to define the size of local problems, and helps the evolution of appropriate solutions by local researchers.^27^ However, creation and sustainability of such comprehensive services is hampered by several problems, the most important being the lack of human resources for healthcare and research.

The major challenges for surgery for cardiovascular diseases in African children include (1) building local capacity and expertise in the diagnosis and management of congenital heart diseases; (2) training local surgeons in the management of conditions that are geographically restricted to Africa, using indigenous techniques that can be applied locally at lower costs; (3) improving the efficiency of equipment and systems for adequate maintenance of equipment; (4) creating infrastructure for the management of and research into cardiovascular diseases in children; (5) creating systems for financial support of the communities served by these infrastructures; (6) addressing the issue of poor local governance and low commitment from policy makers.

The success of the institutional collaborations currently in place, and their acceptance and recognition by the community will hopefully encourage local governments to increase their support, making an effort to become partners with these initiatives while giving enough independence to the projects. Indeed, we think that although individuals or private institutions can start these networks, political support and willingness are crucial to ensure their sustainability in the medium and long term. Finally, the institutions that are running successful programmes must be reinforced as regional referral centres.

## Conclusion

The surgical treatment of cardiovascular diseases in children remains a major challenge in most African countries. Although requiring major investments, surgical programmes must be stimulated, allowing comprehensive management of patients and offering a unique opportunity for research into the particular aspects of geographically restricted diseases, namely the development and testing of approaches to management tailored to the specific conditions of the continent.
